# Supplementary material for: Tracking Immunity: An Increased Number of COVID-19 Boosters Increases the Longevity of Anti-RBD and Anti-RBD-Neutralizing Antibodies
Source: Vaccines (Basel). 2025 Jan 12;13(1):61. doi: 10.3390/vaccines13010061 (PMC11769131; doi:10.3390/vaccines13010061)
Supplement: Supplementary file 1 [file vaccines-13-00061-s001.zip › vaccines-3362091-supplementary.pdf]

Supplemental data for

Tracking Immunity: An Increased Number of COVID-19 Boosters Increases the Longevity of Anti-RBD and Anti-RBD-Neutralizing Antibodies

Ching-Wen Hou [1], Stacy Williams [1], Veronica Boyle [1], Alexa Roeder [2], Bradley Bobbett [1], Izamar Garcia[1], Giavanna Caruth[1], Mitch Magee [1], Yunro Chung [1,3], Douglas Lake [2], Joshua LaBaer [1,4], and Vel Murugan [1*]

[1] Virginia G. Piper Center for Personalized Diagnostics, Biodesign Institute, Arizona State University, Tempe, AZ 85281, USA; chou14@asu.com (C.-W.H.); stacy.adriana.williams@asu.edu (S.W.); vboyle@asu.edu (V.B.); bbobbett@asu.edu (B.B.); izamar.garcia@asu.edu (I.G.); gcaruth@asu.edu (G.C.); mitch.magee@asu.edu (M.M.); yunro.chung@asu.edu (Y.C.); jlabaer@asu.edu (J.L.)

[2] School of Life Sciences, Arizona State University, Phoenix, AZ 85004, USA; ajroeder@asu.edu (A.R.); douglas.lake@asu.edu (D.F.L.)

[3] College of Health Solutions, Arizona State University, Phoenix, AZ 85004, USA;

[4] School of Molecular Sciences, Arizona State University, Phoenix, AZ 85004, USA;

* Correspondence: vel.murugan@asu.edu; Tel.: +1(480)727-0402

Table S1: Seroconversion by race, age, gender, employment status, and the types of vaccines

Variable	Comparison	Anti-RBD Antibody (Access SARS-CoV-2 IgG II)				Anti-NC antibody (Platelia NC total Ab)			
		n	PR	95% CI	P-value	n	PR	95% CI	P-value
Race	White vs Other	368 vs 172	1.00	(0.93, 1.08)	0.93	229 vs 114	0.95	(0.83, 1.09)	0.50
	Asian vs Other	268 vs 172	0.96	(0.84, 1.09)	0.53	134 vs 114	0.94	(0.81, 1.08)	0.35
	White vs Asian	368 vs 268	1.05	(0.92, 1.19)	0.50	229 vs 134	1.02	(0.90, 1.15)	0.74
Age	26-40 vs 18-25	224 vs 373	1.02	(0.91, 1.14)	0.72	130 vs 191	0.91	(0.79, 1.04)	0.16
	41+ vs 18-25	161 vs 373	1.00	(0.85, 1.18)	0.99	84 vs 191	1.07	(0.89, 1.29)	0.47
	41+ vs 26-40	161 vs 224	0.98	(0.86, 1.12)	0.75	84 vs 130	1.18	(0.99, 1.41)	0.06
Gender	Male vs Female	366 vs 442	0.99	(0.94, 1.05)	0.84	211 vs 266	0.97	(0.87, 1.07)	0.52
Employment Status	Student vs Employee	487 vs 321	0.99	(0.85, 1.14)	0.85	278 vs 199	1.12	(0.95, 1.31)	0.17
Vaccine Group#	mRNA vaccine vs Other vaccine	482 vs 326	0.99	(0.88, 1.12)	0.92	270 vs 157	0.91	(0.82, 1.01)	0.07
	Unvaccinated vs Other vaccine	N/A	N/A	N/A	N/A	50 vs 157	1.06	(0.72, 1.55)	0.78
	mRNA vaccine vs Unvaccinated	N/A	N/A	N/A	N/A	270 vs 50	0.86	(0.58, 1.26)	0.44

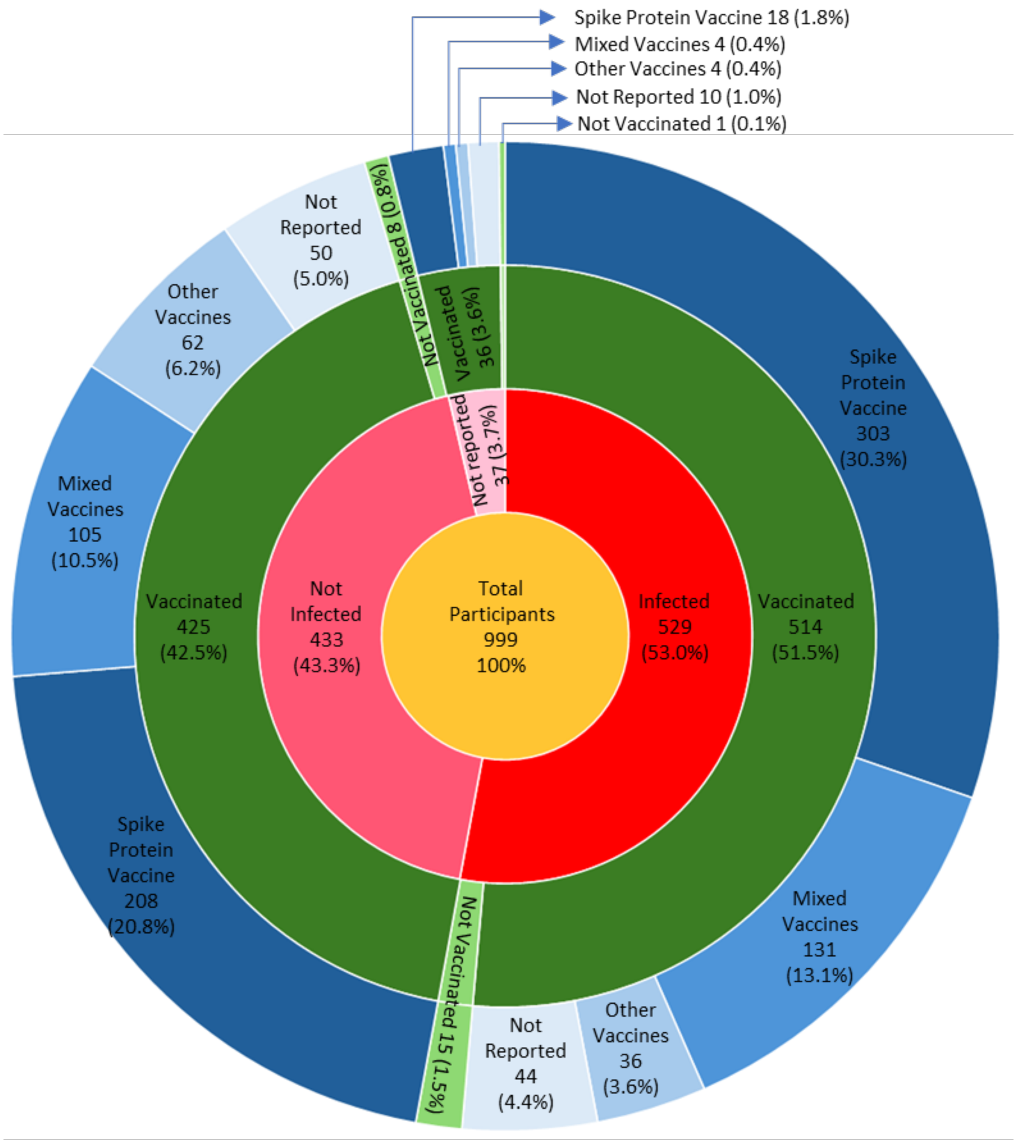

Spike protein vaccine: Pfizer, Moderna, Janssen, and AstraZeneca
Mixed vaccines: Participants received vaccines from different manufacturers
Other vaccine: Vaccines other than Pfizer, Moderna, Janssen and AstraZeneca

Figure S1. Illustration demonstrating the number of participants with self-reported vaccination and infection.

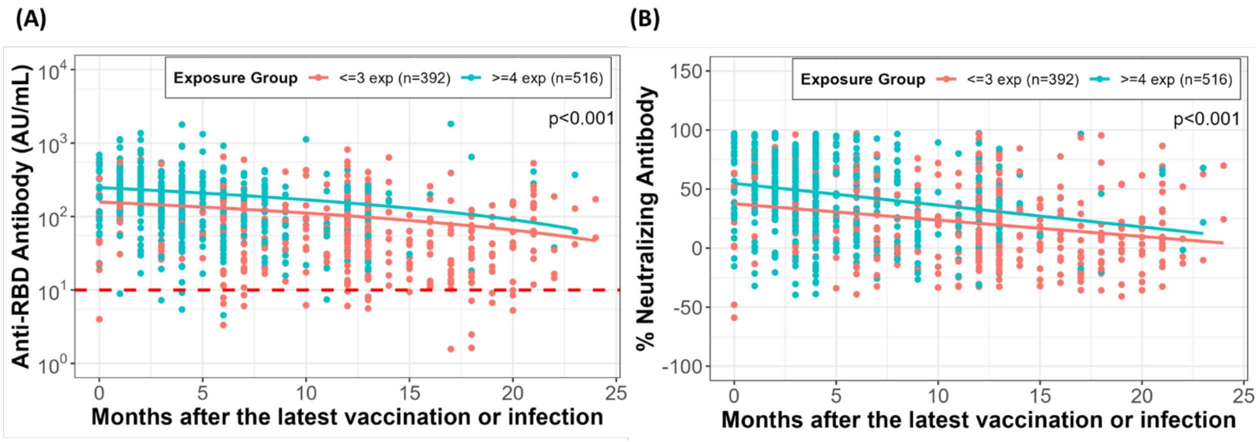

Figure S2. Anti-RBD antibody and neutralizing antibody decay in participants exposed to SARS-CoV-2 antigens through infection or vaccination. (A) Anti-RBD antibody decay (B) Neutralizing antibody decay.

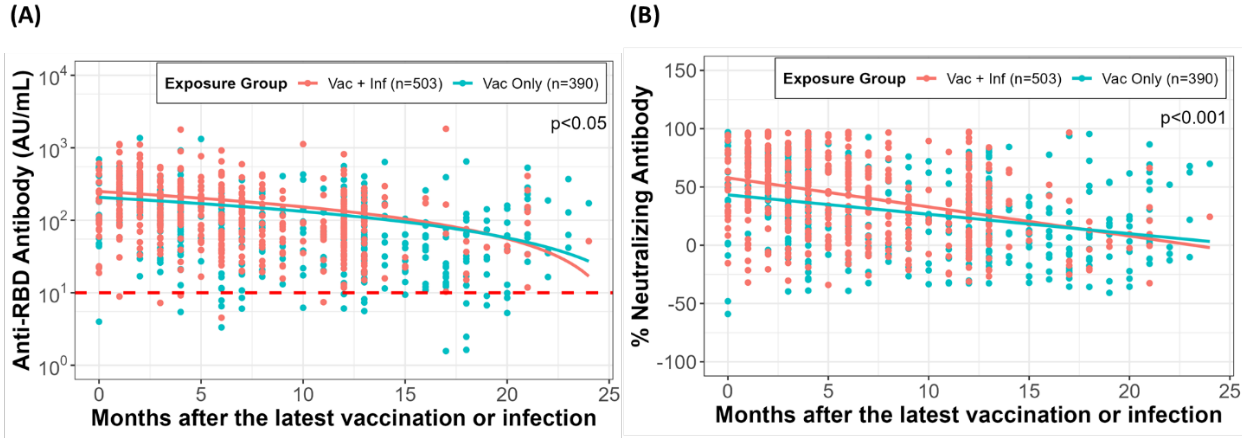

Figure S3. Anti-RBD antibody and neutralizing antibody decay in participants with vaccination and infection compared to vaccination only. (A) Anti-RBD antibody decay (B) Neutralizing antibody decay. Each point represents an individual data measured.
